# Hypersensitivity to Suture Anchors

**DOI:** 10.1155/2013/932167

**Published:** 2013-07-11

**Authors:** Masafumi Goto, Masafumi Gotoh, Yasuhiro Mitsui, Ryo Tanesue, Takahiro Okawa, Fujio Higuchi, Naoto Shiba

**Affiliations:** ^1^Department of Orthopedic Surgery, Kurume University Medical Center, 155 Kokubu-machi, Kurume, Fukuoka 839-0863, Japan; ^2^Department of Orthopedic Surgery, Kurume University, 67 Asahi-machi, Kurume, Fukuoka 830-0011, Japan

## Abstract

Hypersensitivity to suture anchor is extremely rare. Herein, we present a case in which hypersensitivity to suture anchor was strongly suspected. The right rotator cuff of a 50-year-old woman was repaired with a metal suture anchor. Three weeks after the surgery, she developed erythema around her face, trunk, and hands, accompanied by itching. Infection was unlikely because no abnormalities were detected by blood testing or by medical examination. Suspicious of a metallic allergy, a dermatologist performed a patch testing 6 months after the first surgery. The patient had negative reactions to tests for titanium, aluminum, and vanadium, which were the principal components of the suture anchor. The anchor was removed 7 months after the first surgery, and the erythema disappeared immediately. When allergic symptoms occur and persist after the use of a metal anchor, removal should be considered as a treatment option even if the patch test result is negative.

## 1. Introduction

Suture anchors are commonly used, and the literature indicates that they are biologically safe [1–3]. However, loosening, migration, and chondral injury are reported as the most common complications of suture anchors [4]. In this study, we present a case of hypersensitivity to a suture anchor, which is extremely rare.

## 2. Case Report

**Figure 1:**
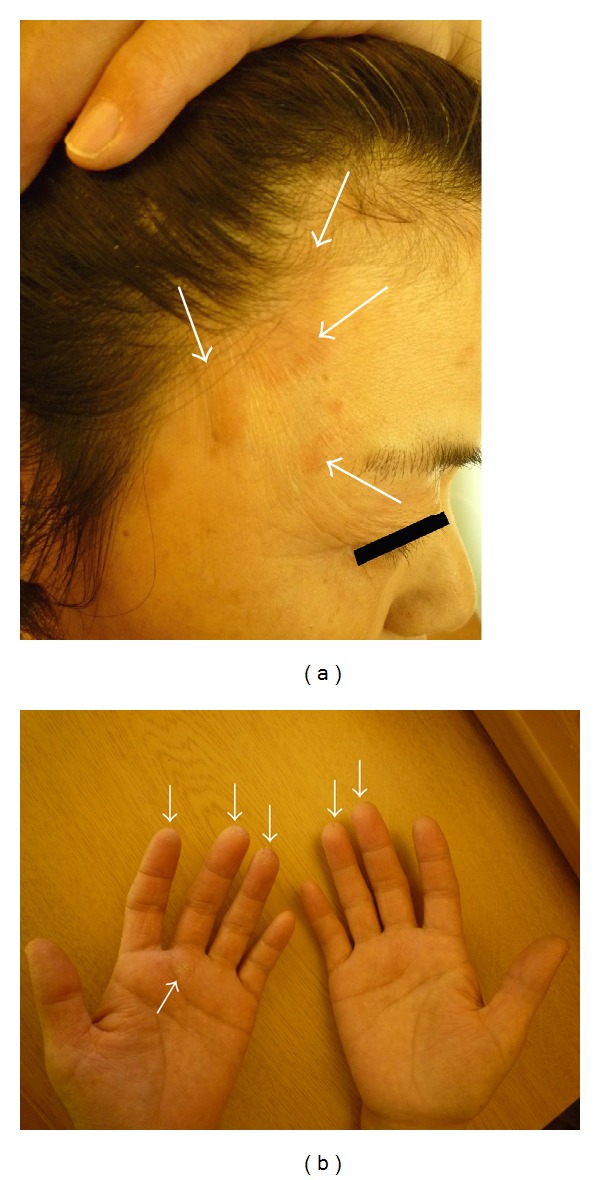
(a) The white arrows indicate erythema around the patient's temple. (b) The white arrows indicate desquamation of the fingertips after the erythema.

**Figure 2:**
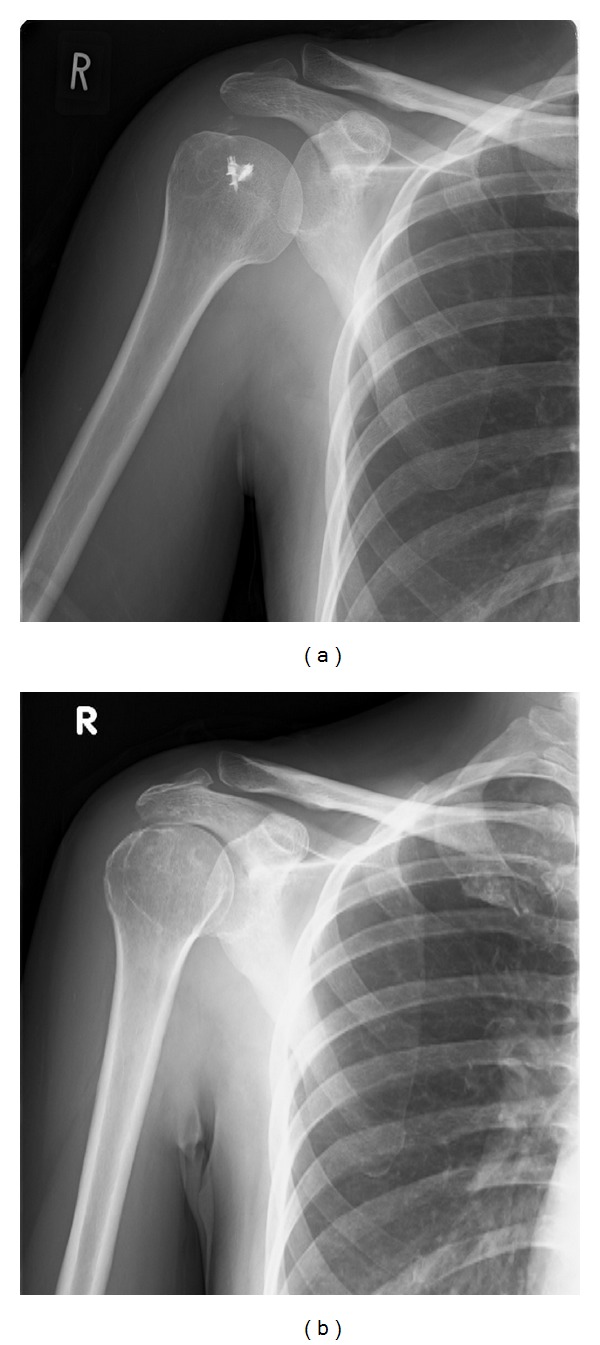
Plain radiograph (anteroposterior view). (a) Before the second surgery. (b) After the second surgery.

A 50-year-old woman experienced calcific rotator cuff tendonitis in her right shoulder. After arthroscopic excision of the calcium, the rotator cuff was repaired with a metal suture anchor (Fastin RC, DePuy Mitek Inc., Raynham, MA). Three weeks after the surgery, she developed erythema around her face, trunk, and hands, accompanied by itching (Figures 1(a) and 1(b)). Findings from plain radiography were normal at that time (Figure 2(a)), and there was no history of allergies, including hypersensitivity to drugs or metals. Although various dermatological treatments such as steroid-containing ointments were used, the symptoms did not improve.

Inflammatory markers, including leukocyte and C-reactive protein, were normal; however, the number of eosinophils was twice the normal values. Suspicious of a metallic allergy, a dermatologist performed a patch testing 6 months after the first surgery. The patient had negative reactions to tests for titanium, aluminum, and vanadium, which were the principal components of the suture anchor.

With the patient's consent, the metal anchor was removed 7 months after the first surgery. During the second surgery, we found that the repaired rotator cuff was well healed and the surrounding cancellous bone had not eroded. After removal of the suture anchor, the rotator cuff was repaired with a nonabsorbable suture (no. 2 Ethibond, DePuy Mitek Inc., Raynham, MA) through the bone tunnel. Pathologic examination of the cancellous bone surrounding the screw revealed no remarkable inflammatory findings (Figure 2(b)).

The erythema and itching disappeared immediately after the second surgery. The patient returned to her previous work without shoulder pain or range-of-motion restrictions.

## 3. Discussion

Although titanium and its alloys are commonly used as medical materials in osteosynthesis and arthroplasty, there have been few reports of hypersensitivity reactions in association with titanium-based suture anchors. A suture anchor used for the arthroscopic rotator cuff reconstruction mainly consists of titanium, which eventually led to a hypersensitive reaction in the present case.

Material reactions to suture anchors were first reported by Chow and Gu [3]. In their case, the patient (a 78-year-old man) developed rashes on different areas of his body, with yellowish drainage 6 to 8 weeks after arthroscopic cuff repair with a metal suture anchor. The second surgery revealed that the bone surrounding the anchors had eroded with substitution of the necrotic tissue. After the second surgery, the patient's symptoms gradually improved. On the basis of these findings, they denied the possibility of infection and concluded that a secondary immune rejection reaction directed against the titanium anchor itself should be considered.

Contrary to the report of James et al., in which pus discharge from the incision line and bone erosion around the anchor was present, there were no drastic changes in our case. In their report, the ends of the anchors were exposed from the greater tuberosity, with the looseness of the sutures holding down the rotator cuff. Thus, this suggests that in addition to hypersensitivity to the anchors, the anchor ends would have impinged on the acromion, causing the aseptic inflammatory reaction, including the pus discharge. Although the apparent reasons for the differences between the previous case and our case remain unclear, the fact that the eczema and itching vanished immediately after anchor removal strongly suggests hypersensitivity to the suture anchor, even though the patch test result was negative.

Peter et al. have reported a similar case to our own: a 35-year-old male patient experienced a first metacarpal fracture on his right hand and presented with eczema a few weeks after osteosynthesis with the use of a pure titanium miniplate and screws. As hypersensitivity to the titanium was suspected, patch testing and lymphocyte transformation testing were performed. The patch testing revealed no negative reactions; however, they found marked hypersensitivity of the patient's cells to titanium in the lymphocyte transformation test. After implant removal, the eczema disappeared immediately. They concluded that the titanium caused an allergic condition, therefore supporting the sequence of events in their case [5]. Lymphocyte transformation testing would have verified the titanium-induced allergic reaction in our case.

In the present report, we demonstrate a case in which a hypersensitive reaction to the metal anchor was strongly suspected. When allergic symptoms continue after the use of a metal anchor, its removal should be considered as a treatment option, even if the patch testing result is negative.
